# Systematic review of sleep disorders in cancer patients: can the prevalence of sleep disorders be ascertained?

**DOI:** 10.1002/cam4.356

**Published:** 2014-11-30

**Authors:** Julie L Otte, Janet S Carpenter, Shalini Manchanda, Kevin L Rand, Todd C Skaar, Michael Weaver, Yelena Chernyak, Xin Zhong, Christele Igega, Carol Landis

**Affiliations:** 1 Indiana University School of NursingIndianapolis, Indiana; 2 Indiana University School of MedicineIndianapolis, Indiana; 3 Indianapolis Department of Psychology, Indiana University-Purdue UniversityIndianapolis, Indiana; 4 University of Washington School of NursingSeattle, Washington

**Keywords:** Cancer, review, sleep, sleep disorder, symptom assessment

## Abstract

Although sleep is vital to all human functioning and poor sleep is a known problem in cancer, it is unclear whether the overall prevalence of the various types of sleep disorders in cancer is known. The purpose of this systematic literature review was to evaluate if the prevalence of sleep disorders could be ascertained from the current body of literature regarding sleep in cancer. This was a critical and systematic review of peer-reviewed, English-language, original articles published from 1980 through 15 October 2013, identified using electronic search engines, a set of key words, and prespecified inclusion and exclusion criteria. Information from 254 full-text, English-language articles was abstracted onto a paper checklist by one reviewer, with a second reviewer randomly verifying 50% (*k* = 99%). All abstracted data were entered into an electronic database, verified for accuracy, and analyzed using descriptive statistics and frequencies in SPSS (v.20) (North Castle, NY). Studies of sleep and cancer focus on specific types of symptoms of poor sleep, and there are no published prevalence studies that focus on underlying sleep disorders. Challenging the current paradigm of the way sleep is studied in cancer could produce better clinical screening tools for use in oncology clinics leading to better triaging of patients with sleep complaints to sleep specialists, and overall improvement in sleep quality.

## Introduction

Sleep is vital to all human functioning and encompasses a complex set of physiological and behavioral processes; disruption in one or more of these processes can lead to many different types of symptoms of poor sleep that can occur singly or in combination. In cancer patients, disturbed sleep is rated the second most bothersome symptom based on cancer and treatment status 1. Sleep problems are known to cause poor healing, increase chances of cancer recurrence, decreased cognitive functioning, decreased work productivity, increased safety issues, medication misuse and abuse, poor relationships, and increased health care costs 2–20.

Poor sleep is a known problem in cancer patients along the treatment trajectory from the point of diagnosis to end of life 21,22. In the United States in 2013, estimates were that there are over 13.7 million people living as cancer survivors, another 1.66 million will be diagnosed with cancer, and more than 580,000 will die of cancer. Poor sleep is known to affect up to 75% of these individuals 23,24. Because of the importance of sleep and the high prevalence of poor sleep in cancer, sleep-related research, and symptom management have been designated as research priorities by the Oncology Nursing Society 25 and National Institute of Nursing Research 26.

Poor sleep in cancer patients and survivors could be attributed to the presence of one or more underlying sleep disorders. Sleep disorders can be classified using two main classification systems, the Diagnostic and Statistical Manual of Mental Disorders-IV (DSM-IV) 27 or the International Classification of Sleep Disorders (ICSD) 28, and they can directly impact health-related quality of life. Sleep disorders can occur singly or in combination and include insomnia, sleep-related breathing disorders, hypersomnia, circadian rhythm disorders, parasomnias, sleep-related movement disorders, isolated symptoms, and other nonspecified disorders 29. Diagnosing specific sleep disorders usually requires a detailed and specialized evaluation, sometimes requiring overnight evaluation of objective measures of sleep. However, it has been reported that cancer patients often do not get referrals to sleep specialists when presenting with chronic sleep complaints. In a recent study of 78 patients with serious insomnia complaints, only four (5%) received a recommendation for formal follow-up or reassessment of the sleep problem 20.

Details of specific sleep disorders should guide intervention(s) since each type of sleep disorder may require a different type of treatment. It is also pertinent to examine how treatment of such sleep disorders fits within the scope of practice for specialty clinics such as oncology. However, the extent to which sleep disorders have been studied in cancer is unclear, even though this information is vital to appropriate assessment and intervention. Therefore, the purpose of this systematic literature review was to evaluate if the prevalence of various types of sleep disorders could be ascertained from the current body of literature regarding sleep in cancer.

## Methods

This was a critical and systematic review over a 2-year time frame of peer-reviewed, English-language, original articles published from 1980 through October 2013. We used PubMed, PsychINFO, CINAHL, and OVID search engines. Key words used for the search included; sleep, sleep disturbances, sleep problems, insomnia, circadian rhythm, restless leg syndrome, sleep apnea, narcolepsy, daytime dysfunction, daytime sleepiness, cancer, breast cancer. Breast cancer was specifically added as a search term to capture the large body of literature from this population on sleep. Limits were human, English language, and adults. Articles were included if: (1) sleep was identified as a primary outcome, a secondary outcome, or a covariable of interest; and (2) articles were focused on cancer diagnosis, treatment, survivorship, or end of life (not prevention). Excluded were review articles, case studies, and abstracts.

Article titles and abstracts were reviewed and screened for the following keywords; sleep, cancer, symptom cluster, menopausal symptoms, correlates of sleep, narcolepsy, circadian, restless leg syndrome, apnea, disturbance, and daytime dysfunction. If the title seemed to fit review criteria, the full-text article was pulled and read to determine relevance to the review. If the article was deemed relevant, information about the article was abstracted onto a paper checklist. Once the final article list was identified, references were checked against the available systematic reviews in the literature to ensure all relevant articles were captured. One reviewer completed data abstraction for all selected articles. A second reviewer randomly checked 50% of the articles for data abstraction accuracy. A kappa statistic was calculated and indicated good agreement between the raters (*k* = 99%), confirming the accuracy of the data abstraction. All abstracted data from the paper checklists were entered into an electronic database. All data entry was double checked for accuracy. Data were analyzed using SPSS (v.20) (North Castle, NY). Descriptive statistics and frequencies were obtained to synthesize the major points of this review.

## Results

**Figure 1:**
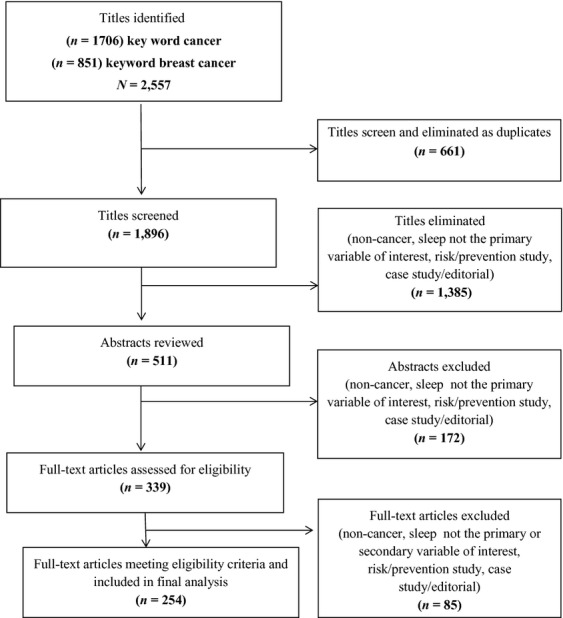
PRISMA diagram for sleep review.

The Preferred Reporting Items for Systematic Reviews and Meta-Analyses (PRISMA) 30 format was used as a guide to develop a search diagram showing the flow for the article retrieval process (Fig.1). A total of 2620 article titles were reviewed. This process resulted in identifying 339 full-text articles that were reviewed.

Of these, 254 articles met criteria to be included in this review. The 254 articles were mainly from the United States (*n* = 145, 57.5%) 3–7,10–12,14,31–162, United Kingdom/Europe (*n* = 43, 16.9%) 8,163–186, and Canada (*n* = 25, 9.8%) 16,187–228. Very few were published during the 1980s (*n* = 4, 1.6%) 78,82,128,135 or 1990s (*n* = 16, 7.1%) 10,14,43,46,50,56,95,104,110,115,121,189,199,208,212,229, with most published in 2000 or later (*n* = 233, 91.3%) 3–8,11,12,15,16,31–42,44–49,51–55,57–75,77,79–81,83–85,87–94,96–103,105–109,111–114,116–120,122–127,129–134,136–188,190–198,200–207,209–211,213–228,230–269. Quantitative descriptive studies were most common (*n* = 186, 73.6%), 3–8,11,12,14–16,31,32,34,37,38,40,43–46,48–50,54–63,67–76,78,80,82,84–101,103–108,110–116,118–123,126,129,130,132,133,135–140,142–146,148,150,151,154,155,158–160,162,163,165,168–171,173–177,181–185,188,191,194–199,202–209,211,213–215,217–221,223,224,227–233,235,237,239–243,245–249,251–255,257,259,260,263,265–269 followed by intervention studies (*n* = 58, 22.8%), 35,36,38,39,41,42,47,51–53,64,65,77,81,83,102,109,113,117,124,127,128,131,134,141,147,149,153,156,157,161,164,167,172,178–180,186,187,189,190,193,201,210,216,222,225,226,234,238,244,250,256,258,261,262,264 qualitative studies (*n* = 7, 2.8%) 66,79,152,166,192,200,212, and mixed-methods studies (*n* = 2, 0.8%) 10,33.

### Description of studies

Study samples primarily included patients with a mix of different types of cancers (*n* = 104, 40.9%) 3,4,8,10,14,35,36,45,47,48,50,52,58,62,66,68,69,72,75,78,80–82,84,88,89,93,95,98–100,103–105,111,113,115–117,120–122,127–130,143,145,149,161,162,164–170,174,181,182,184,187,189–192,194–196,198,203,206,208,212,215,216,218,221,223,224,227–229,231,232,234,236,238,240–243,245,247,248,253,254,259,261,264,266–268 or patients with breast cancer (*n* = 102, 40.2%), 5–7,11,12,15,16,31,32,34,37–44,46,49,51,55–57,59,61,63–65,73,76,77,79,83,85–87,91,92,94,96,97,101,102,106,108,112,118,119,123–126,131,132,134,136–141,148,151,154–160,163,172,173,175,176,178–180,183,186,188,193,200–202,210,214,220,222,225,226,230,233,239,249–251,256,263,265 with fewer studies focused on another single cancer diagnosis (*n* = 48, 18.9%) 33,38,53,54,67,70,71,74,107,109,110,114,133,135,142,144,146,147,150,152,153,171,185,197,199,204,205,207,209,211,213,217,219,235,237,244,246,252,255,257,258,260,262,269. Of the latter, studies focusing on lung cancer were most common (*n* = 14) 70,71,74,109,110,135,144,152,197,204,205,235,246,257. The total number of participants with cancer appeared as high as 3343, although there was duplication where data were reported in more than one manuscript. The number of participants in the control groups (including cancer and noncancer) evaluated in studies was 1607. Only 90 studies (35%) 3,7,11,34–36,38,39,43,46,48,50–53,58,63–65,69–71,74,77,78,85,93–95,101,102,106,109,113,115,117,122,125,128,129,132–135,137,141,145–147,149,153–157,161,163,178,179,187,188,190,191,193,194,204,214–217,219,224,225,229,234,236,237,240,244,245,247,250,255,256,258,261,262,264 included a comparison group in the study design, with most comparison groups being subjects with cancer.

### Sleep-specific characteristics of studies

Methodological characteristics of the 254 articles are shown in Table1. The time points for assessing sleep were varied but mainly focus on accessing sleep problems during and postcancer treatment. Most articles reviewed focused on sleep as a concurrently occurring or clustered variable with either fatigue or one or more other symptoms (59%). 3,5–7,12,14–16,32,33,35,37–49,51–54,56–62,67–69,71,73–77,79–86,88,89,92,93,96,97,101,102,104–108,110–114,116,118,122,124–127,129–131,133,134,136–150,154–160,162–164,167–170,173,178,179,183,185,188,191,193,195,198,200–203,207,208,212,214–216,218,220–230,234,241,243–245,247,248,250–254,256–259,261,263–269.

**Table 1:** Methodological characteristics of reviewed articles

Characteristic	*N* (%) *n *=* *254
Type of cancer	
Mixed	104 (40.9)
Breast	102 (40.2)
Other	48 (18.9)
Time point	
Pretreatment	25 (9.8)
During treatment	76 (29.9)
Posttreatment	72 (28.3)
Palliative care	17 (6.7)
Mixed	49 (19.3)
Type of study design	
Descriptive	187 (73.6)
Intervention	58 (22.8)
Qualitative	7 (2.8)
Mixed methods	2 (0.8)
Priority of sleep	
Primary alone	83 (32.7)
Concurrent with fatigue	23 (9.1)
Concurrent with cluster	147 (57.9)
Sleep term defined	
Yes	50 (19.7)
No	204 (80.3)
Formal classifications of sleep disorders used	
Yes	26 (10.2)
No	228 (89.8)
Etiology of sleep problem provided	
Yes	64 (25.2)
No	190 (74.8)
Subjective measure of sleep	
PSQI	81 (31.9)
Single item from standardized form	46 (18.1)
No subjective measure	28 (11.0)
Other	99 (39.0)
Objective measure of sleep	
Actigraphy	5 (20.9)
Polysomnography	16 (6.3)
None	185 (72.8)
Included biomarkers of sleep	
Yes	18 (7.1)
No	236 (92.9)
Assessed medication use (prescription and over-the-counter)	
Yes	64 (25.2)
No	190 (74.8)
Body mass index reported	
Yes	44 (17.3)
No	213 (82.7)
Noncancer comorbidities reported	
Yes	41 (16.1)
No	213 (83.9)
Discipline of author(s)	
Nursing	62 (24.4)
Mixed	99 (39.0)
Medicine	48 (18.8)
Psychology	25 (9.8)
Other or not listed	20 (8.0)
Country of origin of authors	
United States of America	146 (57.5)
United Kingdom/Europe	43 (16.9)
Canada	25 (9.8)
Year published	
1982–1989	4 (1.6)
1990–1999	18 (7.1)
2000–2013	232 (91.3)
Discipline of author(s)	
Nursing	62 (24.4)
Mixed	99 (39.0)
Medicine	48 (18.8)

Interventions studied	*N* (%)*n* = 54
Cognitive-behavioral therapy	23 (42.6)
Pharmacologic	6 (11.1)
Other	25 (46.3)

When selecting participants for studies, potentially important inclusion and exclusion criteria for sleep were rarely addressed. For example, only 19.7% (*n* = 50) 35–41,48,49,163,187,233–235,61,63,69,77,87,90,91,99,108,109,115,122,129,136,138,140,141,151,160,161,172–174,178–181,189,192,204,217,221,255,266 of the studies reviewed excluded for previous sleep disorder or current treatment of sleep disorders.

The majority of studies did not include a definition of sleep or sleep problems, did not classify sleep disorders, and did not discuss the etiology of the sleep problems studied. Only 26 (10.2%) 8,36,40,58,64,65,77,97,98,108,109,115,161,172,173,176,178,179,182,190,192,209,210,216,231,240 studies used a formal diagnostic classification system within the study design. Thirteen of these 26 studies used the DSM-IV (*n* = 13) 36,65,97,108,109,161,176,190,192,210,216,231,240. The remaining 13 articles used a combination of the DSM and ICSD (*n* = 8), ICSD alone (*n* = 4), or a classification used by the American Society of Dentist Anesthesiologists (*n* = 1). Those using diagnostic classification systems used them as a mechanism to verify eligibility for studies specifically looking for primary insomnia. No other specific details from these studies were provided such as frequency of other possible sleep findings that could be used to establish some prevalence information.

In addition, sleep was primarily assessed using self-report and not objective measures. The most common standardized measure used was the Pittsburgh Sleep Quality Index (*n* = 81), a valid and reliable instrument. The most common approach was a single item or multiple items from a larger standardized or investigator-generated questionnaire. For objective measures, wrist actigraphy was the most common approach, with polysomnography only used in 16 studies. Wrist actigraphy is typically used more often due to the expense of polysomnography, which often requires an overnight stay in a hospital or clinic for evaluation.

Few studies reported assessing biomarkers such as ferritin, which plays a role in the etiology of restless leg syndrome. In addition, few studies reported concurrent medication use, particularly medications that could help or hinder sleep. Finally, few studies reported body mass index (important to diagnosing the potential for sleep apnea) or noncancer comorbidities that could hinder sleep. Potentially important exclusion criteria such as poor performance status, psychiatric illnesses, cognitive impairment, and anemia were also often not reported or not used.

Interventions were a part of only 33, or 13%, of the total studies reviewed. The most common interventions tested were formal or investigator-generated behavioral treatments for insomnia. Other interventions included mostly nonpharmacotherapy interventions such as acupuncture, yoga, relaxation, and exercise. Only five studies tested pharmacotherapy for treatment. The efficacy of these interventions for cancer patients has been assessed and can be found on the Oncology Nursing Society Putting Evidence into Practice website (www.ons.org).

## Discussion

The major conclusion of this review is that the prevalence of overall particular types of sleep disorders in cancer cannot be ascertained using currently available literature. This is true across studies using mixed cancer samples and larger studies that focus specifically on populations of breast and lung cancer patients and survivors.

The main reason we cannot ascertain prevalence of specific sleep disorders relates to the conceptualization and operationalization of poor sleep. Our review indicates that studies have focused on measuring general symptoms of poor sleep rather than on the underlying sleep disorders. Symptoms of poor sleep include a decreased number of hours of sleep (sleep duration), increased number of minutes to fall asleep (sleep latency), nighttime awakenings (sleep disruptions), and inability to function fully during the day without naps (daytime dysfunction) 14,270. These symptoms are consistent with insomnia and can occur singly or in combination, affecting overall sleep quality and daytime function 271–274. These symptoms help to define poor sleep but are common to more than one sleep disorder 21. In the reviewed literature, we found that the terminology for symptoms of poor sleep is often used interchangeably with that for specific sleep disorders which therefore are not fully assessed.

This imprecise conceptualization of sleep has led to narrowly focused interventions being diffusely targeted to symptoms, rather than focused and specific to one or more sleep disorders underlying those symptoms. Although some interventions for sleep in cancer have shown possible efficacy 25, the majority of these studies are too targeted to undefined subtypes of insomnia and therefore are not generalizable to the many patients who may have other types of sleep disorders 113,178. This makes translating these interventions into oncology practice difficult.

To ascertain prevalence of the various types of disorders, we must change the current method for studying sleep in persons with cancer, which has focused on using single- or multi-item scales to assess and classify symptoms of poor sleep 17,21,275. A total of 33 different subjective sleep measures were used across studies, with little consistency among those measures although the Pittsburgh Sleep Quality Index, used in 32% of the studies, was the most common. These measures neglect the larger issue of assessing specific types of sleep disorders underlying symptoms (e.g., trouble falling asleep and staying asleep, nighttime disturbances) that are common to several different sleep disorders. Particular types of sleep disorders require different interventions, yet most intervention studies target undefined subtypes of insomnia as a single, underlying sleep disorder. This is problematic because there are 11 different subtypes of insomnia and not all are responsive to the same intervention 28.

Based on reports from clinical practice, patients can experience more than one type of sleep disorder concurrently, which influences how sleep is treated. For example, one seminal study found that 40% of breast cancer survivors had symptoms suggestive of both insomnia (undeclared subtypes) and a sleep-related movement disorder (e.g., restless leg syndrome) 8. In addition, although sleep apnea has long been assumed to be relatively uncommon in women (<9%), one study of midlife women without cancer who reported symptoms of poor sleep found that 53% met criteria for sleep apnea 276. This supports the finding that postmenopausal women have sleep apnea at the same prevalence rate as men 277. These findings also suggest that the next logical step in sleep research for cancer patients is to correctly identify the prevalence of distinct types of sleep disorders in patients reporting symptoms of poor sleep so that appropriate clinical assessment tools and interventions can be developed and tested by oncology and general health-care providers. Perhaps this type of clinical assessment could be started in relation to one type of cancer such as in breast cancer patients and then translated to other cancer diagnoses or other chronic illnesses for testing.

### Limitations of this review

Conclusions from this systematic review should be tempered in light of some limitations. First, our review focused on English-language articles, which may have excluded some important information about sleep disorders in cancer. Second, although every attempt was made to identify all pertinent articles, it is possible that some were missed, which also might have affected our conclusions.

### Implications for practice

Sleep disorder prevalence data would be helpful for future intervention development. One place to begin is determining how symptoms are being addressed in the clinic visit. The role of oncology health practitioners in delineating complaints of sleep problems remains a challenge. With several formal practice guidelines available, the reality of clinic visits is that addressing symptoms is determined by a multitude of variables. During treatment there are sometimes other cancer-specific symptoms that are obvious contributing factors to acute sleep problems. For example, patients with end-stage lung cancer often have breathing problems that mimic sleep apnea or metastatic pain that is not controlled, and in this context pharmacological approaches may be appropriate. However, if such obvious problems are not present during survivorship it is unclear if oncology practitioners have the appropriate assessment tools to decide whether to make referrals to sleep specialists who could formally diagnose sleep disorders. Although practice guidelines can provide a global understanding of how to treat a patient with sleep complaints, future interventions for sleep need to involve educating about and implementing the use of short assessment tools for oncology patients and their providers as a basis for more effective treatments in the clinic setting and/or appropriate referrals for further testing. An assessment tool would need to be brief and target the common sleep disorders such as restless leg syndrome, sleep apnea, and possibly narcolepsy (although less frequently found in the general population). This tool would need to be sensitive enough to warrant appropriate referral to sleep medicine specialists for further diagnostic evaluation. Integrating into practice could be in the form of a screening tool that integrates into the electronic medical record to reflect current clinic practices. Future interventions should also be translatable to smaller community settings where specialized oncology clinics are not readily available.

For research there are several considerations moving forward. Revising common inclusion and exclusion criteria used in research studies is required to better reflect the complexity of sleep. Although specific inclusion and exclusion criteria for studies is largely dependent on the type of research question(s) being addressed, specific questions that identify possible symptoms of common sleep disorders for both descriptive and intervention research would better reflect the influence the fact that people tend to have more than one type of sleep problem. For behavioral intervention work this is especially crucial because having undiagnosed sleep disorders can have a negative impact on the efficacy findings of those studies. In addition, refining current measurements of sleep that better assist in detangling the common sleep complaint is required. Specifically, screening all research subjects using a brief screening form for sleep disorders would better identify the prevalence of specific sleep disorders across the cancer trajectory. Providing this type of information would better inform if different questionnaires were needed based on the type of cancer being studied based on the prevalence in that population.

## Conclusion

Although the literature, especially in the past 10 years, has raised awareness that poor sleep is problematic throughout the cancer trajectory, the prevalence of particular types of sleep disorders in cancer remains unclear. This is likely due to the primary focus on studying symptoms of poor sleep and not characterizing the underlying sleep disorders in cancer patients and survivors. This review indicates that little is known about the prevalence of specific sleep disorders in cancer, which hampers the ability to fully understand (1) how to triage clinical assessments of sleep complaints by all levels and types of health-care practitioners that have contact with cancer patients and survivors and (2) how best to intervene for patients presenting in specialty clinics such as oncology with sleep complaints. Future studies should challenge the current research paradigm that focuses on describing and intervening narrowly on symptoms of poor sleep. Sleep screenings are needed that are tailored to facilitate better triage, referral for further assessment when appropriate, and more effective interventions that take into account the multiple types of sleep disorders.

## Conflict of Interest

None declared.
